# Methodological pluralism for better evaluations of complex interventions: lessons from evaluating an innovation platform in Australia

**DOI:** 10.1186/s12961-022-00814-5

**Published:** 2022-01-28

**Authors:** J. Bailie, F. Cunningham, S. Abimbola, A. Laycock, R. Bainbridge, R. Bailie, K. Conte, M. Passey, D. Peiris

**Affiliations:** 1 The University Centre for Rural Health, The University of Sydney, 61 Uralba Street, Lismore, NSW 2480 Australia; 2 The School of Public Health, The University of Sydney, Sydney, Australia; 3 Menzies School of Health Research, Charles Darwin University, Brisbane, Australia; 4 The George Institute for Global Health, The University of New South Wales, Sydney, Australia; 5 School of Health, Medical and Applied Sciences, Central Queensland University, Cairns, Australia; 6 The School of Public Health, De Paul University, Chicago, USA

**Keywords:** Innovation platforms, Developmental evaluation, Principles-focused evaluation, Network analysis, Collaborations, Utilization-focused, Systems thinking, Complex interventions

## Abstract

Complex interventions, such as innovation platforms, pose challenges for evaluators. A variety of methodological approaches are often required to build a more complete and comprehensive understanding of how complex interventions work. In this paper, we outline and critically appraise a methodologically pluralist evaluation of an innovation platform to strengthen primary care for Aboriginal and Torres Strait Islander Australians. In doing so, we aim to identify lessons learned from the approach taken and add to existing literature on implementing evaluations in complex settings, such as innovation platforms. The pluralist design used four evaluation approaches—developmental evaluation, principles-focused evaluation, network analysis, and framework analysis—with differing strengths and challenges. Taken together, the multiple evaluation approaches yielded a detailed description and nuanced understanding of the formation, functioning and outcomes of the innovation platform that would be difficult to achieve with any single evaluation method. While a methodologically pluralist design may place additional pressure on logistical and analytic resources available, it enables a deeper understanding of the mechanisms that underlie complex interventions.

## Background

Innovation platforms are complex interventions [1–3] and, as such, present challenges for their evaluators [4–6]. They are characterized by actors from diverse disciplines and stakeholder groups collectively problem-solving, exchanging ideas from different perspectives, and sharing expertise to generate new knowledge and solutions that could not be achieved by one discipline, or stakeholder group, alone [7, 8]. Innovation platforms differ from other types of collaborations in several ways [7–9]. Firstly, they incorporate a wider network of members operating at multiple levels of a system and in different roles within it. Secondly, they embrace the concept of “boundary spanning” by bringing in members from other sectors to assist in developing solutions to challenges [9]. And, finally, they have continuous reflection, learning and adaptation as central design elements to support innovation [3, 7]. Despite the importance of evaluating these collaborations, there are a few critical appraisals of the different approaches that can be taken in such evaluations.

To build a complete and comprehensive understanding of how complex interventions work requires various evaluation approaches [4, 6, 10–12]. The value of this methodological pluralism, which in its simplest form denotes diversity, is seen in its ability to provide a more holistic and textured analysis, allowing for a complete understanding of the situation, and in its potential to redress the limitations inherent in any single method [11, 13–17]. Methodological pluralism thus refers to an approach which applies more than one methodology and method, and at times, more than one epistemological stance [14]. However, using pluralist methodologies raises several challenges, including assembling an evaluation team with the skills and experience across multiple evaluation approaches and methods; acquiring the resources to implement data collection using a variety of strategies; and undertaking the analysis and synthesis of collected data using multiple and diverse approaches [18].

In this paper, we outline and critically appraise a methodologically pluralist evaluation of an innovation platform in Aboriginal and Torres Strait Islander (hereafter referred to respectfully as Indigenous Australian, acknowledging cultural and historical diversity) primary healthcare (PHC). The paper first gives the setting of the innovation platform and then describes its evaluation and the four evaluation approaches employed: developmental evaluation [3]; principles-focused evaluation [19]; network analysis [20]; and framework analysis [21]. We then identify the lessons learned from undertaking a methodologically pluralist evaluation, and issues to consider when planning and conducting evaluations of complex interventions such as innovation platforms. In doing so, we provide an opportunity for others to learn from our experience, extending the literature on evaluating complex interventions. This paper is based on the critical reflections of the authors, many of whom were part of the evaluation team.

## Evaluation setting: an innovation platform

Indigenous Australians have extraordinary cultural strength, adaptability and resilience, and yet continue to experience poorer health outcomes and shorter life expectancy compared to other Australians [22]. The reasons for this are complex but are rooted in the pervasive legacy of colonization—land dispossession, displacement, disempowerment, social and economic exclusion, and ongoing racism [22, 23]—and centuries of government paternalism and neglect, which Indigenous Australians continue to challenge and work to redress.

Established in November 2014, the Centre for Research Excellence in Integrated Quality Improvement (CRE-IQI) aimed to improve Indigenous health outcomes by embedding and strengthening continuous quality improvement (CQI) in PHC [20, 24]. The CRE-IQI, funded for 5 years by Australia’s National Health and Medical Research Council (NHMRC) as an innovation platform [7], fostered and built on relationships between Indigenous community-controlled health organizations, government-managed PHC centres, research institutions, government health departments and key regional support organizations (e.g. health councils) to embed system-wide CQI. Indeed, some of its stakeholders had already worked together for more than 15 years in participatory CQI research and development with Indigenous PHC [20].

Continuing the spirit of the collaboration from previous years, the innovation platform of the CRE-IQI was an “open collaboration” that encouraged and welcomed new members. Within the scope of “integrated quality improvement” [25], it collaboratively developed and refined both research priorities to address key stakeholder needs and a set of principles to govern practice [19]. The innovation platform enabled PHC practitioners and policy-makers to articulate knowledge gaps and work with researchers and health sector stakeholders on relevant research topics [7]. It also encouraged new collaborations by sharing information, open seed-funding calls to develop projects and promoting collaborative research.

By participating in biannual face-to-face meetings, stakeholders could build relationships, progress project development and research translation, and share the project methodologies, findings and outcomes of their research. Similarly, masterclasses were hosted around each of the biannual meetings with a focus on enhancing the skills and knowledge of innovation platform members on a variety of topics related to CQI. Online monthly research capacity-building seminars were also held.

Further details about how the CRE-IQI operated as an innovation platform [3, 7, 24], results from the respective evaluative approaches [7, 19–21, 26] and research findings of the CRE-IQI are published elsewhere [21, 24, 26]. In Box 1 we summarize the CRE-IQI research findings, engagement and impact [20, 21, 24].

**Table:** 

Box 1: CRE-IQI key research findings, engagement and impact [20, 21, 24] *Key research findings from the CRE-IQI *[24] 1. CQI has been widely accepted and applied in Indigenous health services and in PHC settings, with some resulting improvements in clinical care, service systems and the social determinants of health 2. Indigenous leadership and participation in PHC services and research improves the quality of care delivered 3. Clinical and non-clinical health outcomes can be improved by using evidence-based CQI tools and processes 4. Access to accurate and timely data across the scope of practice is essential for CQI in comprehensive PHC and for informing and driving health service, intersectoral and community action 5. Priorities have been identified for strengthening PHC systems to achieve large-scale health improvement for Indigenous people *Engagement and impact of the CRE-IQI* Research translation • 90 peer-reviewed publications [20] (450+ citations and 185,000+ downloads) • 7 policy/parliamentary submissions; 27 research and technical reports; 81 conference presentations • 26 CRE-IQI newsletters, with an average of 70 individual opens per newsletter Collaboration • 72 different organizations had contributing authors on CRE-IQI peer-reviewed publications, with 263 individual authors [20] • 47 different lead authors from 22 different organizations • Strong connections between CRE-IQI members with 43% of CRE-IQI members collaborating with people they did not know before their involvement in the CRE-IQI [24] • Coauthorship of publications shows an increasing core-periphery structure of the CRE-IQI, as opposed to a single dominant organization (this points to a more collaborative network) [20] • 10 biannual meetings to bring together collaborators in 4 different locations across Australia, with 120 individuals attending at least one biannual meeting • $31,998,410 leveraged in collaborative research grants Research capacity-strengthening • 24 students affiliated (PhD, masters, undergraduate placements) • 31 research capacity-strengthening seminars held • 28% of peer-reviewed publications had a student/programme officer as lead author, and 58% of publications had at least one student/project officer as an author [20] • 16 masterclasses enabled researchers and service providers to access professional development on topics identified by CRE-IQI members, with 166 individuals attending at least one masterclass • $2,600,920 leveraged in scholarship and fellowship funding Indigenous leadership and participation • 62% of peer-reviewed publications had at least one Indigenous author [20]; 67% of presentations had at least one Indigenous author [24] •46% of individual attendees at biannual meetings were Indigenous and/or representing and Indigenous organization • Participation by Indigenous people and organizations increased from 27% in the first biannual meeting to 44% in the final 2019 meeting •Established co-leadership arrangements between Indigenous and non-Indigenous researchers • 39% of individual attendees at masterclasses were Indigenous and/or representing an Indigenous organization	

## Evaluation model

One of the primary aims of the CRE-IQI was to monitor and evaluate the CRE-IQI as an innovation platform. The overall evaluation goal was to study the formation, functioning and outcomes of the CRE–IQI as an innovation platform. The evaluation had the following objectives:

1. To refine the formation, functioning and outcomes of the innovation platform by supporting continuous reflection, rapid learning and adaptation.
2. To identify the mechanisms and contextual factors that enable innovation platforms to have a positive impact on Indigenous PHC systems.
3. To assess the development of, and change in, innovation platform collaborators over time.
4. To generate new knowledge on, and approaches to, evaluating innovation platforms.

The effective conduct of the evaluation was one of the primary responsibilities of the CRE-IQI research fellow (evaluation) (JB) (herein referred to as “evaluation fellow”). This position had dual responsibilities related to coordination and implementation of the evaluation, and CRE-IQI project management. An evaluation working group provided oversight and guidance for the evaluation. The group chaired by an Indigenous researcher/evaluator comprised researchers with specific evaluation skills and responsibilities within the CRE-IQI. Initially, the evaluation working group was virtual, but as the work progressed it was agreed that more regular focused meetings were needed to bring together the evaluation strands, streamline the data collection, implement a group analysis of emerging data, and provide evaluation project management oversight. From mid-2017, fortnightly teleconferences were facilitated by the evaluation fellow and six-monthly face-to-face evaluation specific meetings held.

**Fig. 1:**
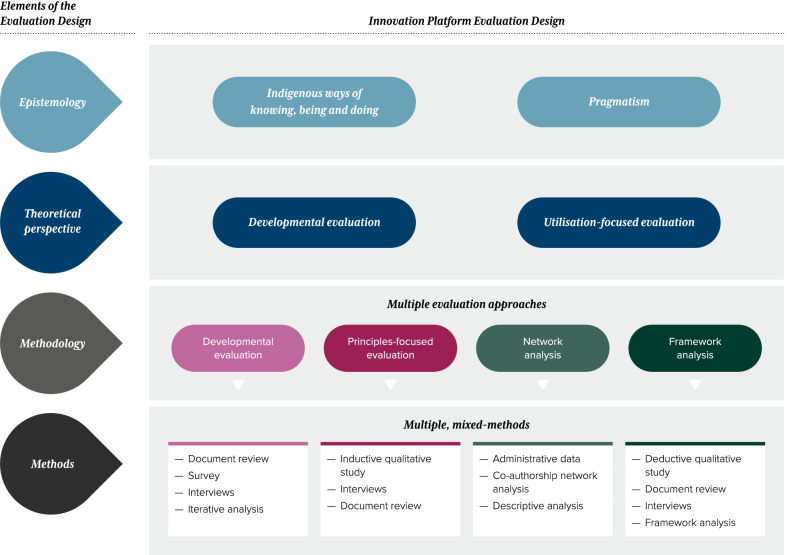
Key elements of the evaluation design of the innovation platform. ^1^ Drawing on Crotty’s [27] four elements of research design and Lemire et al.’s [28] evaluation tree

In designing the key evaluation components of the innovation platform, the evaluation working group drew on Crotty’s [27] four elements of research design and Lemire and colleagues’ [28] “evaluation tree”, modified from Christie and Alkin’s [29] “evaluation theory tree”. These components are outlined in Fig. 1 and further discussed in relevant sections of this paper.

The *epistemology* layer is concerned with what informs our perspectives [27]. As shown in Fig. 1, the evaluation of the innovation platform had an Indigenous perspective, which valued and centred Indigenous knowledge systems [30, 31] by taking a strengths-based approach and adopting an emergent interactive design. The evaluation was guided by a set of co-created principles, for example, respecting the past and present experiences of Indigenous peoples, working in partnership, and ensuring Indigenous leadership and direction of research in all stages of the process [19]. The evaluation also took a pragmatic philosophical approach [13, 32] based on the proposition that researchers should use the philosophical and/or methodological approach that works best for the particular research question and research context [33–35]. Pragmatism embraces the use of a plurality of methods in which the focus is on the situation and opportunities that emerge, rather than on adherence to a fixed design [17, 18, 36]. Moreover, it encourages evaluation questions to search for useful and actionable answers [36]. Grounded in Indigenous ways of knowing, being and doing and coupled with a pragmatic philosophical approach, we adopted a constructivist perspective, which assumes that neither data nor theories are discovered but rather are constructed based on the shared experiences of researchers and respondents [30, 31].

The *theoretical perspective* layer relates to how the evaluation will be used, by whom and for what purpose [28]. Following the pragmatic epistemology, our theoretical perspective was driven by the evaluation use and purpose, which we conceptualized as both “developmental” and “utilization-focused” (see Fig. 1) [32, 37, 38]. Developmental purpose aligned with the need for innovation platforms to have a mechanism for continuous reflection, learning and adaptation to support innovation [3]. To this end, we collected and interpreted data, developed and implemented change strategies, evaluated how well they worked, and repeated the cycle with different sets of data and feedback, thereby informing and supporting the innovation platform’s formation, functioning and outcomes.

A focus on utilization was paramount, not least because many of our end-users were participants in the innovation platform. As evaluators, we facilitated a learning and decision-making process that focused on how the evaluation’s findings and experiences would be used to encourage its ownership by users and create momentum for them to implement the findings [32, 38].

The *methodology* layer in Fig. 1 details the methodologically pluralist design, which included the following evaluation approaches: developmental evaluation [3], principles-focused evaluation [19], network analysis [20] and framework analysis [21]. The *methods* layer describes the specific methods employed for each evaluation approach.

Given the integrated nature of methods and use in evaluation practice [28], it is inevitable that there is congruency and flow between the *theoretical perspective* and *methodology* layer. For example, developmental evaluation is placed on more than one layer because of the primacy of the approach in the use of the evaluation, that is, to inform the ongoing formation, functioning and outcomes of the innovation platform, and as an important methodological approach.

Utilization-focused nor developmental evaluation advocate for a standardized methodology or a priori evaluation objectives [38]. Rather, situational responsiveness guides an emergent process between the intended users of the evaluation and the evaluator to select the most appropriate approach for their needs and to adapt it reflexively as circumstances and evaluation objectives evolve [32]. Given the focus on “learning and adaption” in this approach, it was neither possible nor appropriate to detail a priori evaluation methods, objectives or outcomes [32]. This is in contrast to other evaluation approaches which aim to answer a priori research questions or which focus on refining programme theory within predefined configurations (e.g. realist evaluation).

**Fig. 2:**
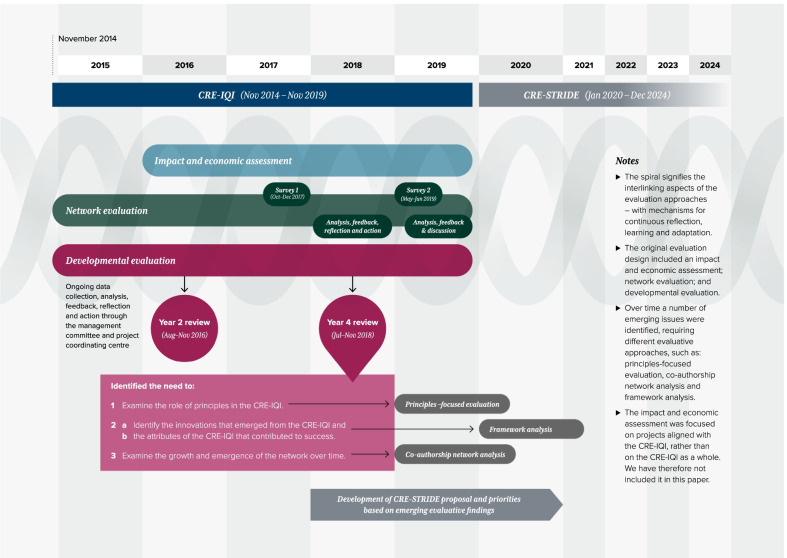
Timeline of the CRE-IQI evaluative activities, demonstrating linkages between evaluative approaches. *CRE-IQI* Centre for Research Excellence in Integrated Quality Improvement; *CRE-STRIDE* Centre for Research Excellence in Strengthening Systems for Indigenous Health Care Equity

In addition to the four evaluation approaches outlined in Fig. 1 and Table 1, we conducted an impact and economic evaluation. As the impact and economic evaluation was of specific research projects associated with the innovation platform, they are reported in separate publications [39, 40]. Figure 2 depicts the evaluation of the CRE-IQI over time and the linkages between the evaluations. This figure is further discussed in relevant sections of this paper.

**Table 1:** Evaluation design of the innovation platform and key findings

Objectives of the evaluation	Methodology—evaluation approaches	Rationale for the chosen methodology and methods	Implementation, data collection and analysis	Brief overview of evaluation findings
Objective 1: To refine the formation, functioning and outcomes of the innovation platform by supporting continuous reflection, rapid learning and adaptation	*Evaluation approach 1:* Developmental evaluation [3]	•To inform the formation, functioning and outcomes of the collaboration, we focused on the use of data to inform ongoing decision-making, reflection and adaptation—e.g. operations of the innovation platform, work programmes and future directions •Developmental evaluation embraces innovation, complexity and systems thinking. Innovation platforms are complex systems and have continuous reflection and adaptation as design elements	•Administrative project records provided *ongoing* intelligence on the innovation platform development and context, e.g. minutes from the management committee and biannual stakeholder meetings; publications, posters and conference papers; attendance lists and evaluations of biannual meetings, masterclasses and research capacity-strengthening teleconferences; research project applications; and student projects •Major contributions were the CRE-IQI Year 2 and Year 4 Reviews •Interviews as part of Year 4 Review (*n* = 28) •Further round of interviews to explore emergent themes from Year 4 Review (*n* = 36) •Analysis and feedback, iterative and ongoing	•Developmental evaluation was well suited to innovation platforms, where there is a developmental purpose, innovation niche and complexity •Adjustments made to the operation of the innovation platform based on the evaluative feedback included adding an Indigenous researcher to the innovation platform leadership team; targeting resources for policy and parliamentary submissions; implementing collaborative processes to identify and refine research priorities; and discussing principles of the innovation platform at the start of regular six-monthly meetings •We used opportunistic and planned iterative cycles of reflection and analysis to understand how, and how well, the innovation platform was functioning and meeting its goals and how it could be adapted in rapid time to function more effectively
Objective 2: To identify the mechanisms and contextual factors that enable innovation platforms to have a positive impact in Indigenous PHC systems	*Evaluation approach 2:* Principles-focused evaluation [19]	•Emerged from feedback from innovation platform members, as part of the developmental evaluation, on the importance of the principles in guiding our work. From this, we agreed to a principles-focused evaluation to address objective 2 •To examine in depth how the principles we developed to underpin the governance of the collaboration were implemented and their expected outcomes	*Data collection* •Purposive sampling to capture a diversity of views from interviews with innovation platform members (*n* = 35) by two researchers •Reflective summary generated after each interview •Document review of administrative project documents, results from developmental evaluation, publications, etc. *Data analysis* •Data inductively coded and categorized into strategies, outcomes and conditions •Member-checking processes included presenting early findings at innovation platform meetings; comparing, contrasting and seeking consensus of findings between c0-authors; and triangulation with findings from document reviews	•The principles were viewed as an integrated whole, with overlap but mutually reinforcing, that would enable us to navigate complexity and conflict •Implementation of the principles occurred through five strategies: honouring the principles; being dynamic and adaptable; sharing and dispersing leadership; collaborating purposefully; and adopting a culture of mutual learning •Outcomes included increased Indigenous leadership and participation; the ability to attract principled and values-driven researchers and stakeholders; and the development of trusting and respectful relationships •The conditions that facilitated the implementation of the principles were collaborating over time; increasing the number of Indigenous researchers; and taking an “innovation platform” approach •Given that the focus of innovation platforms is on empowering local actors to solve problems collaboratively, and as lessons on their use accumulate, the findings from our study suggest that there is scope to be more explicit about the principles governing them and to embed, constantly monitor and reflect on their role within innovation platforms
	*Evaluation approach 3:* Framework analysis [21]	•To gain an understanding of the elements that enabled the innovation platform •Emerged when it became evident that the evaluation working group needed to find an appropriate way to answer critical questions about the attributes of an innovation platform	*Data collection* •Purposive sampling to capture a diversity of views from innovation platform members (*n* = 35) by two researchers •Document review of publications and reports from the innovation platform *Data analysis* •Framework analysis using a taxonomy as framework. [41] Data were deductively coded to the four primary elements—innovation, communication, time and social system. Though primarily a deductive qualitative approach, we remained “nimble to emerging attributes”, and this application enabled us to identify emergent attributes not encompassed within the taxonomy •Findings compared and contrasted, and a consensus process from authors undertaken through multiple reviews, triangulation of findings and discussions	•The long history of working together enabled trusting relationships, a collective identity and a foundation for new people to join •Time was identified as a crucial element •Innovation was stimulated by bringing people together to learn, share ideas and solve problems, with Indigenous participation and leadership at the core of the research agenda-setting •The innovation platform outputs exceeded 92 peer-reviewed publications; 81 conference presentations; 27 research and technical reports; 26 newsletters, 16 masterclasses; 31 research capacity-strengthening webinars; 24 students (PhD, masters and undergraduate) •The innovation platform had 18 research projects with investigators from 27 different organizations and $31,998,410 in collaborative research grants •There was an ongoing need to focus on increasing the engagement of and leadership by Indigenous and health service stakeholders
Objective 3: To assess the development of, and change in, innovation platform collaborators over time	*Evaluation approach 4:* Network analysis [20]	•To understand why and how the collaboration has grown and changed over time •Coauthorship approach emerged from the developmental evaluation and discussions with members who wished to explore the growth and change in membership, in particular, its Indigenous representation	*Coauthorship network analysis* *Data collection* •Peer-reviewed journal articles and books published by authors from the network *Data analysis* •Co-author networks across four phases of the network (2002–04; 2005–09; 2010–14; 2015–19) were constructed based on author affiliations •Social network analysis methods •Descriptive characteristics included organization types, Indigenous representation, gender, student authorship and thematic research trends *Social network analysis* *Data collection* •Surveys of those defined as active members of the innovation platform at two time points (2017 and 2019) *Data analysis* •Social network analysis methods	*Coauthorship network analysis* •Publications accelerated when the collaboration changed to an “innovation platform”, which coincided with a broader thematic focus and an increased number and diversity of participating organizations •This expansion occurred largely due to the cumulative effect of building trust and relationships over time, including the development of a comprehensive data set on CQI in Indigenous PHC for use by all stakeholders •Network analyses indicated a core/periphery structure of organizations connected to each other, rather than a network structured around a single central organization •Increased productivity was associated with increased authorship diversity and a decentralized network, suggesting these may be important factors in enhancing research impact and advancing the knowledge and practice of CQI in PHC •Despite improvements over time, further work is needed to address inequities in both female and Indigenous authorship *Social network analysis* [24, 26] •There was more sharing of knowledge and collaborating between those who had prior knowledge of each other. However, 48% also reported sharing and 37% collaborating with people of whom they had no prior knowledge. This shows both a broadening of relationships and a sharing of knowledge not only with existing partners but also new ones •In addition, 36% of sharing occurred outside immediate collaborative partnerships, indicating good network support •Further reporting is expected on how well the innovation platform worked as a collaborative network over time
Objective 4: To generate new knowledge on and approaches to evaluating innovation platforms	The application of the multiple evaluation approaches listed above speak to this objective	As listed above	As presented in this manuscript	As presented in this manuscript

Table 1 briefly outlines the rationale for the evaluation approaches, their implementation, respective key findings and how they link with the objectives of the evaluation. What is described in Table 1 emerged over time because of reflection and learning. For each evaluative approach there is a publication that has more detailed background, rationale, methods and findings [3, 19–21].

### Evaluation approach 1: Developmental evaluation to inform the continuous reflection and adaptation of the innovation platform

The developmental evaluation, reported in full elsewhere [3, 26], had several strengths. Firstly, the methodology embraced situations with a developmental purpose, innovation niche and a focus on complexity, which is highly apposite for innovation platforms. Secondly, the collaborative data analysis approach provided immediate, useable feedback to engage innovation platform members in co-creating responses to findings. For example, feedback was received through biannual meetings and other mechanisms about the need to strengthen engagement with policy-making processes. In response, training was provided on engaging with policy-makers, and resources were directed into writing targeted policy and parliamentary submissions that drew on the research of the innovation platform. Thirdly, we observed that evaluating the innovation platform developmentally allowed for the acquisition of new knowledge and skills through multiple interactions between stakeholders.

The developmental evaluation encouraged and allowed the generation of evidence in rapid time through a flexible, situationally tailored evaluation design. It provided the space to identify new evaluation questions and, therefore, new evaluation approaches to emerge, for example, the principles-focused evaluation and coauthorship network analysis. Importantly, it was congruent with the CQI focus of the innovation platform itself, such as collecting and interpreting data, developing, implementing and evaluating change strategies and then repeating the cycle. Thus, innovation platform members were already familiar with this way of thinking, and this likely increased their receptivity to this style of feedback and action planning.

### Evaluation approach 2: Principles-focused evaluation to explore how the innovation platform functioned

Principles-focused evaluation is a relatively new and emerging direction in evaluation, in which principles are the evaluand [41]. Operation of the innovation platform was governed by a set of collaboratively developed principles such as Indigenous leadership and direction in all stages [19]. These principles were critical to defining and setting the course for the collaboration, that is, the primary way of navigating the complexity of the collaboration. As previously mentioned, the principles-focused evaluation [19] arose in direct response to the developmental evaluation findings, in which members of the innovation platform identified a need for further exploration of how the principles were implemented in its operations and what outcomes were produced as a consequence of using the principles. There was keen interest and engagement from innovation platform members in the novel evaluative approach in which the development and application of the principles themselves are the evaluand.

We used an inductive qualitative approach that was appropriate for Indigenous settings and for tackling questions about which there was little prior research [30]. The evaluation also gave “voice” to members of the innovation platform through a series of interviews and iterative analytical processes.

### Evaluation approach 3: Widening our focus by using network analysis to assess collaboration and knowledge generation

Findings from the developmental evaluation and the principles-focused evaluation pointed to the over 15-year history of the collaboration (commencing in 2002) on which the innovation platform was built, and the primacy of this positive history of working together in enabling its effectiveness [20]. Unexpectedly, we needed to look wider than the planned social network analysis, at the big picture, or “zoom out” to examine the growth and emergence of the innovation platform; specifically, how the CRE-IQI was addressing its vision of strengthening capacity, equity and membership diversity. Network analysis [20], with its good visualization tools, offered us a feasible strategy for widening our evaluation focus which would allow us to capture deep collaboration through multiple authorship. As publications are available in publicly accessible databases and previously collated for other reporting purposes, there was minimal burden on other evaluative activities of collaboration members. We recognize, however, that coauthorship is only one indicator of collaboration, and it may not reflect our many other collaborative outputs, such as grant submissions and conference presentations.

### Evaluation approach 4: Framework analysis to understand how and why the innovation platform functions

The framework analysis emerged from discussions within the evaluation working group and among innovation platform members, on the need to gather perspectives on how the innovation platform functions and to identify the drivers of its success. In this approach, we mapped primary data (interviews with innovation platform members) and secondary data (publications and reports related to the innovation platform as a whole) to a taxonomy that characterized the attributes (innovation, communication, time, social systems) of the innovation platform [21, 42]. In doing so, we produced a new theorization that could shed further light on and extend lessons from both our research and completed evaluations. The approach was primarily a deductive qualitative approach, though we remained “nimble to emerging attributes”, and this application enabled us to identify emergent attributes not encompassed within the taxonomy.

## Insights and lessons learned from our evaluation approach

### Using different approaches enabled a complex systems perspective, generating a more detailed and textured evaluation

From the outset, it was clear that no single approach would achieve all the evaluation objectives. Having multiple evaluation approaches and methods supported a complex systems perspective and is congruent with calls by Indigenous scholars for system science approaches to address complex issues [30]. It enabled us to examine and identify individual mechanisms and their interconnections that supported the desired functioning and operation of the innovation platform while also providing a view of the system as a whole and the collective outputs produced. Furthermore, multiple evaluation approaches enabled us to acquire a more comprehensive and textured account of the innovation platform’s formation, function and outcomes. For example, the principles-focused evaluation allowed us to inductively develop an understanding of how the innovation platform’s guiding principles led to increased Indigenous leadership and participation, and, in turn, the coauthorship network analysis demonstrated the growth and change in Indigenous participation by examining coauthorship patterns.

### An evaluation working group and an embedded evaluation fellow enabled streamlining of data collections, course corrections and decision-making

Dedicated resourcing for an evaluation working group and the appointment of a part-time evaluation research fellow helped to (1) coordinate evaluative activities and streamline data collection opportunities; (2) make necessary course corrections by providing a forum to discuss emergent issues and options, while remaining focused on the overall evaluation goals; and (3) provide a forum to discuss proposed methodological approaches and interim findings. Importantly, this group also guided decisions about data use and storage and protocols for acknowledgement of data sources and authorship [43].

Consistent with a developmental evaluation approach [3, 44], the evaluation fellow was an embedded team member rather than a traditional external evaluator [45]. As the position required dual responsibilities of both project management and implementation of evaluation, this allowed the evaluation fellow to formally participate in the management committee, evaluation working group and other relevant meetings. Attendance at core governance and operational meetings facilitated an understanding of emergent issues and the need for timely action among key decision-makers. This embeddedness meant that any changes to the innovation platform’s direction and evaluation—based on insights, learnings and critically reflective conversations between the evaluation working group and innovation platform management and members—could be expedited as needs arose. Being alert to the potential for positivity bias as an embedded evaluator meant that we sought to ensure there were processes in place to enhance the credibility of findings. Strategies undertaken included (1) the inclusion of two researchers to undertake data collection; (2) highly participatory analysis and interpretation in which researchers not actively engaged in the CRE were included in the analysis team; and (3) use of a variety of data sources to triangulate findings.

The evaluation fellow had a long-standing history of working with innovation platform members on previous research projects and collaborations and an in-depth understanding of CQI and PHC. This background knowledge of the context and existing relationships with end-users catalysed engagement with the evaluation. In other situations, with an evaluator less familiar with the field and/or the evaluation participants, more time would likely be required to conduct a formal situational analysis to understand the context in which the innovation platform exists and to ensure the evaluation design takes this into account.

### The active involvement of “users” in the evaluation while judiciously avoiding evaluation fatigue was key to success

Experience points to the importance of identifying and involving “end-users” of the evaluation, which, in our case, included innovation platform members such as health service providers, researchers and policy-makers. An example of this was the presentation of emergent findings from the developmental evaluation’s Year 4 Review [26] to the CRE-IQI management committee, evaluation working group and the broader network at the biannual meetings. The findings were further synthesized and prioritized during these interactions, and collaborative strategies to address them identified. The active engagement of users in these collaborative analysis processes and discussions to make sense of emergent findings enabled early action and early acquisition of new knowledge rather than waiting for a final report or publication. For example, early findings from the principles-focused evaluation identified the importance of explicitly promoting the shared values and principles of the innovation platform. On discussion with innovation platform members of these early findings, a review of further opportunities to promote the principles was discussed, and it was agreed that the principles were to be applied as criteria on all “seed-funding” applications to develop research.

Given the focus on involving end-users there is, however, a risk of evaluation fatigue if the activities are not well coordinated and perceived as meaningful to participants. Enthusiasm for the involvement of end-users must also consider their primary work responsibilities and demands on their time. For example, in the innovation platform, many of the members were busy health service providers, and some balanced dual clinician/researcher roles. Opportunities for generating engagement included maintaining a focus on innovation platform members’ needs and learning rather than the evaluation itself; being mindful of the capacity of users when planning the collection and analysis of evaluation data; collecting data at one point for multiple purposes; and provision of routine updates and collaborative analysis processes at management committee and scheduled biannual meetings.

### Leveraging data sources for multiple purposes created efficiency gains in data collection efforts

Given our concerns of evaluation fatigue and to limit the burden of evaluation for Indigenous people [30], we proactively looked for opportunities to use existing and practical data sources (i.e. routinely collected data) for multiple purposes and to maximize the output of data collection efforts, rather than continuously collecting new primary data for each evaluation sub-study. For example, we drew on existing collated lists of publications required for project reporting for use in the coauthorship network analysis to understand the growth and emergence of the innovation platform. A further example is the use of existing publications and reports produced by the evaluation of the innovation platform as secondary data for the framework analysis. Thus, while pluralistic methods require more data collection and effort, taking advantage of the existing synergies between the four design frameworks and using practical data sources reduced some of the burden and assisted with a systems thinking approach to explore the complexity of the innovation platform.

### Balancing the need for an emergent evaluation that responded to changing circumstances while remaining focused on the overall evaluation goals and objectives

Methodological pluralism enabled us to respond promptly to the “emergent” nature of a complex system. The findings from the developmental evaluation [3] were important determinants of the subsequent design of the principles-focused evaluation [19], network analysis [20] and framework analysis [21] (Fig. 2). The downside of being responsive to emergent issues is the risk of distraction by interesting but less important issues. Therefore, remaining focused on the goals and objectives of the overall evaluation while valuing flexibility was important. The regular evaluation working group meetings were instrumental in this regard, allowing us to strike a balance between the flexibility required to adapt rapidly to emergent findings and evolving stakeholder needs, and the availability of evaluation resources.

### The co-creation of evaluative knowledge was deeply relational, engaged and underpinned by principles of practice

The Indigenous context we were working in required evaluative knowledge to be co-created with CRE-IQI members. At the core of the “all teach, all learn” motto of the CRE-IQI is the valuing of Indigenous cultures, knowledge and expertise alongside Western research and knowledge—it embodies the value placed on mutual learning [46].

Over time, the CRE-IQI and the evaluation had increasing leadership and participation of Indigenous people, in response to evaluative feedback and subsequent focused and deliberate strategies to achieve this. At the outset, the evaluation did not explicitly state that we were being guided by Indigenous ways of knowing, being and doing. Rather we adopted the “all teach, all learn” motto [46] and were guided by an agreed set of principles of practice [19]. As outlined above, these included Indigenous leadership and direction of research, a partnership approach and respect for the experiences of Indigenous peoples. Using a strengths-based approach, ensuring we were contextually responsive, implementing systems and relational approaches, and an emergent, interactive design supported the operationalization of the principles [19]. There were many conversations amongst CRE-IQI members about what an Indigenous way of working would be and how it would look, as we worked to progress these over time. These conversations may not have taken place, and concerns about Indigenous participation and leadership may not have been raised or given high priority, without the continuing focus on principles of practices and the relational aspects of the CRE-IQI. Meaningful engagement with Indigenous people must occur early through codesign and be sustained throughout the evaluation to co-produce actionable knowledge.

### The commitment of leadership to the developmental evaluation enabled evaluation resourcing, innovation and adaptation

Highly collaborative, methodologically pluralist evaluations are resource intensive, requiring the evaluation team to encompass a wide range of skills and experiences. Because it is unlikely that any single evaluator would have sufficient methodological diversity to tackle all evaluation elements, we needed to strike a balance between what was practically feasible in terms of the resources, time and skills of the evaluation team, and the scientific rigour needed to address the evaluation’s questions.

Reflecting the commitment to undertaking a comprehensive evaluation, resources were budgeted at the grant submission stage for the evaluation (e.g. the evaluation research fellow), supportive structures (e.g. the evaluation working group) and research operations to support collaboration throughout the evaluation (e.g. participatory data analysis). This underscores the need for substantial leadership commitment to the evaluation, not just in terms of resourcing but also in being flexible and open to making changes when required. Leadership commitment to the developmental evaluation and its findings supported the innovation and adaptation of both the evaluation and the innovation platform.

Sufficient time was needed for the participatory analysis and synthesis of findings, and for feeding back preliminary findings from the different evaluation approaches. This feedback proved to be especially important, because some of the final products (i.e. publications) could not be completed until after the innovation platform funding period. Fortunately, we were successful in securing funding for the next 5-year iteration of the innovation platform—through an Indigenous-led Centre for Research Excellence in Strengthening Systems for Indigenous Health Care Equity (CRE-STRIDE). This allowed us to share our learnings and final findings, a process that will in turn inform the evaluation of CRE-STRIDE [20, 47]. In Table 2, we have summarized recommendations for evaluators based on our experience of taking a methodologically pluralist approach to evaluating a complex intervention.

**Table 2:** Recommendations to optimize the benefits of evaluations of collaborations using pluralistic approaches

Ensure that leaders are willing to invest resources in the evaluation to allow it to be undertaken within an adequate time frame, and that leaders are open and flexible to making changes when required.
Assemble an evaluation team with a variety of evaluation expertise and negotiate scope to contract specific methodological expertise as required.
Engage evaluators with high-level facilitation skills to engage and sustain participation.
Use an embedded evaluator to optimize the evaluator’s ability to engage stakeholders in the evaluation and ensure findings are translated into practice.
Keep the overall goal of the evaluation in mind and reflect on the goal regularly when considering emergent and responsive approaches to evaluation findings.
Consider evaluation approaches that allow for “zooming in” on details, but also on “zooming out” to see the big picture and the interconnections within the system.
Be alert to possibilities for maximizing data collection opportunities and coordinate evaluation activities in a way that will avoid evaluation fatigue of collaboration members.
Take advantage of synergies and use of routinely collected data sources where possible to reduce the burden of collecting new data for each evaluation approach.
Enthusiasm for the involvement of end-users in the evaluation must be tempered with clear definitions of who they are and an understanding of the demands on their time.
Create space for reflection and provide flexibility for new user perspectives and new questions to emerge, with the evaluation team or management group offering a forum for this to occur.
Include opportunities in the evaluation plan for reflection on the experience of using pluralist methodologies and on whether methodological changes need to be made.
Include opportunities in the evaluation for feedback to and from stakeholders, e.g. when results from each method are available and at the end of the evaluation for input to integrate the findings.

## Conclusion

A methodologically pluralist evaluation of an innovation platform to improve Indigenous health generated different and complementary insights that would be difficult to achieve with a single-methodology evaluation. Application of the multiple evaluation approaches in this study yielded a detailed description and nuanced understanding of innovation platforms as an “emergent” complex system. While a methodologically pluralist design may place additional pressure on logistical and analytic resources available, it enables a deeper understanding of the mechanisms that underlie complex interventions. Attending to complexity in the design and implementation of the evaluation requires ways of working that are thoughtful, planned and relationally driven.

## Data Availability

Not applicable.
